# Surface Versus Bulk State Transitions in Inkjet-Printed All-Inorganic Perovskite Quantum Dot Films

**DOI:** 10.3390/nano12223956

**Published:** 2022-11-10

**Authors:** Thilini K. Ekanayaka, Dylan Richmond, Mason McCormick, Shashank R. Nandyala, Halle C. Helfrich, Alexander Sinitskii, Jon M. Pikal, Carolina C. Ilie, Peter A. Dowben, Andrew J. Yost

**Affiliations:** 1 Department of Physics and Astronomy, University of Nebraska-Lincoln, Lincoln, NE 68588, USA; 2 Department of Physics, State University of New York-Oswego, Oswego, NY 13126, USA; 3 Department of Chemistry, University of Nebraska-Lincoln, Lincoln, NE 68588-0304, USA; 4 Department of Electrical and Computer Engineering, University of Wyoming, Laramie, WY 82071, USA; 5 Department of Physics, Oklahoma State University, Stillwater, OK 74078, USA; 6 Department of Physics, Pittsburg State University, Pittsburg, KS 66762, USA; 7 Oklahoma Photovoltaic Research Institute, Oklahoma State University, Stillwater, OK 74078, USA

**Keywords:** inkjet-printed perovskites, surface termination, CsPbBr_3_, surface states, bulk states, trap states

## Abstract

The anion exchange of the halides, Br and I, is demonstrated through the direct mixing of two pure perovskite quantum dot solutions, CsPbBr_3_ and CsPbI_3_, and is shown to be both facile and result in a completely alloyed single phase mixed halide perovskite. Anion exchange is also observed in an interlayer printing method utilizing the pure, unalloyed perovskite solutions and a commercial inkjet printer. The halide exchange was confirmed by optical absorption spectroscopy, photoluminescent spectroscopy, X-ray diffraction, and X-ray photoemission spectroscopy characterization and indicates that alloying is thermodynamically favorable, while the formation of a clustered alloy is not favored. Additionally, a surface-to-bulk photoemission core level transition is observed for the Cs 4d photoemission feature, which indicates that the electronic structure of the surface is different from the bulk. Time resolved photoluminescence spectroscopy indicates the presence of multiple excitonic decay features, which is argued to originate from states residing at surface and bulk environments.

## 1. Introduction

Hybrid halide and all-inorganic halide perovskite materials have attracted significant attention in the research community over the past decade [1,2,3,4,5,6,7]. The increased attention is due to their attractive material properties, such as long carrier lifetimes [8,9], long diffusion lengths [8,10], excellent device power conversion efficiency [11,12,13], and band gap tunability [14,15,16,17]. Both the hybrid and inorganic perovskites have shown some promise towards a number of potential applications, such as lasers [18,19,20,21,22], photodetectors [23,24,25], high energy radiation detection [26,27], and of course in photovoltaics [6,28,29]. Although the hybrid halide perovskites show overall higher device power conversion efficiency, >22% [30], when compared to the all-inorganic halide perovskites, >13% [12], the hybrid halides suffer from light sensitivity/degradation and material instability due to environmental conditions [31]. The Pb-based inorganic halide perovskites have an advantage over the hybrid halide perovskites because they show enhanced chemical stability and are overall more robust to environmental conditions [6,32,33,34]. When compared to traditional silicon-based photovoltaic technology, the hybrid and all-inorganic perovskites have an advantage, as they can be synthesized as high quality inks for low cost inkjet printing and the printed films retain the attractive performance features of the inks after printing [9,35,36,37,38].

There have been some major issues with the halide-based perovskites, specifically, optimizing film quality [39], reducing the material instability in laboratory and extreme environments [40], and ion migration-induced device degradation [41], to name just a few. An area of particular interest due to the troublesome nature of surfaces relates to defects and their role in promoting the exciton recombination process [42]. Many techniques, such as interface engineering [43] composition control via precursor stoichiometry [44] doping strategies [42], and organic additive methods [45] have been utilized in an attempt to prevent parasitic defects in optical applications by passivating these high probability recombination sites. The origins of the recombination process can be difficult to pinpoint and often presents contention in the literature. Despite the challenges, there has also been great progress as several methods to synthesize these nanoparticles have been created, such as inkjet printing [9,35,36,37,38], solvo-thermal synthesis [46], and the hot-injection method [15] etc.

In photovoltaic and laser-based technologies it is important to have high device performance with low fabrication costs. The use of photoactive inks in inkjet printing provides a low cost fabrication method with the ability to print high quality thin films in atmospheric conditions [38,47,48]. Additionally, inkjet printing can be used to rapidly prototype or combinatorically determine optimal materials combinations and simultaneously act as a steppingstone to other large scale printing techniques, such as screen printing.

An attractive characteristic of these perovskites is the ease of which the bandgap can be tuned via small changes in the precursor stoichiometric ratios, it can also be tuned through halide exchange, making it an archetypal material for inkjet printing. As such, it is reasonable to assume two different single halide-based inks can be combined, both pre- and post- printing, in order to print custom mixed stoichiometric halide perovskite films, if alloy formation represents a thermodynamic sink that is sufficiently favorable to overcome barriers to interdiffusion.

Here we report the interlayer anion exchange in layer-by-layer inkjet printing of pure perovskite solutions. The printed films exhibit the extraordinary alloying of the two perovskites, which affects the luminescence and is vitally important should the materials find application in display technologies. We investigated the formation of an alloy of a bromide with an iodide to form a CsPbBr_3−x_I_x_, as the efficiency of mixed-halide CsPbIBr_2_ quantum dot solar cells has been reported to show significant improvements [49]. Additionally, a surface-to-bulk core level shift in the electronic structure is observed, which gives a novel insight into the multiple excitonic decay processes for these materials.

## 2. Materials and Methods

For a detailed procedure on the quantum dot ink synthesis, the reader should refer to earlier works [9,37]. After synthesizing the quantum dot powders, they were converted to inks for printing by dissolving, separately, 280 mg of CsPbBr_3_ and CsPbI_3_ powders in 25 mL of cyclohexane (Sigma-Aldrich, St. Louis, MO, USA). The CsPbBr_3−x_I_x_ ink solution was prepared by combining a 1:1 mass ratio of CsPbBr_3_ and CsPbI_3_ ink solutions, known as direct mixing. Mixed inks (CsPbBr_3−x_I_x_) and pure inks (CsPbBr_3_ and CsPbI_3_) were printed on indium tin oxide (ITO)-coated polyethylene terephthalate (PET) flexible substrates (Sigma-Aldrich, St. Louis, MO, USA) or amorphous glass substrates. In a study by Hu et al. [49], annealing was found to reduce vacancy defects, and the inkjet printing process intrinsically is an annealing deposition method, as there is an abrupt temperature increase in an inkjet printer head.

X-ray diffraction (XRD) was employed to investigate the crystal structure for all printed films using a Rigaku Smartlab diffractometer (Rigaku Corporation, Akishima-shi, Tokyo, Japan) equipped with a Cu-Kα X-ray source with a wavelength of 1.54 Å. XRD is complemented by X-ray photoemission spectroscopy (XPS) to further establish the composition of the photovoltaic printed films. XPS was performed with a SPECS Phoibos 150 hemispherical analyzer (SPECS Surface Nano Analysis GmbH, Berlin, Germany) using a non-monochromatized Al-K_α_ X-ray radiation and a pass energy of 15 eV. All XPS was performed at room temperature in an ultra-high vacuum chamber with a chamber pressure better than 5.0 × 10^−10^ mbar. Transmission electron microscopy (TEM) and select area electron diffraction (SAED) were performed on a JEOL JEM-2100 scanning transmission electron microscope (JEOL USA, Inc., Peabody, MA, USA) at an accelerating voltage of 200 kV.

The optical absorption and photoluminescent properties of the quantum dot inks, single layer, and inter-layer printed films were measured using an Ocean Optics HR4000CG-UV-NIR high resolution spectrometer, combined with an Ocean Optics DH-2000-BAL Deuterium-Halogen light source (Ocean Insight, Orlando, FL, USA). Time resolved photoluminescence (TRPL) measurements were carried out at room temperature, on increasing thicknesses of CsPbBr3 single-layer printed films, using an ultrafast laser equipped with a Ti:Sa amplifier system that generates 4 mJ pulses at 800 nm with 100 fs pulse width and a repetition rate of 1 kHz. A portion of the 800 nm pulse is sent to an optical parametric amplifier to generate a 310 nm, 100 fs laser pulse. The sample PL was collected in a backscattering orientation with a high numerical aperture 90° off-axis parabolic mirror. The PL spectra were measured with an Avantes CCD spectrometer (Avantes USA, Lafayette, CO, USA) using a 325 nm long-pass filter to block the pump signal from entering the spectrometer. The measured PL spectra were corrected using a tungsten halogen lamp as a calibration source.

## 3. Results and Discussion

### 3.1. Structural and Optical Properties of Inks and Printed Films

**Figure 1:**
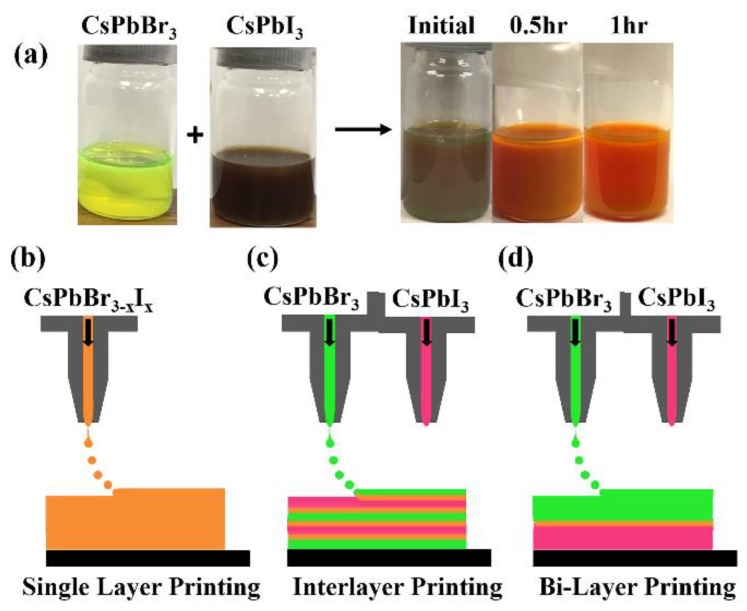
(**a**) (left) the image of pure CsPbBr_3_ (yellow) being added to CsPbI_3_ (brown) inks (right); these inks are directly alloyed in solution. The schematic of the printing methods employed where (**b**) is the single-layer method, (**c**) is the inter-layer method and (**d**) is the bi-layer method.

The direct mixed perovskite (CsPbBr_3−x_I_x_) inks obtained from mixing the two pure CsPbBr_3_ and CsPbI_3_ quantum dot solutions are shown in Figure 1a. This alloying of the perovskite nanoparticles occurred in a solution at room temperature without any temperature treatment or other further efforts to aid the process (like stirring). Figure 1a shows how the color of the quantum dot ink mixture changes from brown to orange with time, a signature of the material resolving into an alloy of intermediate band gap. The color change is believed to be due to an anion exchange, whereby the Br and I ions migrate within the pure perovskite solutions to form a new mixed perovskite [50]. The alloying process takes approximately 30 min to 1 h; however, for larger nanoparticles the alloying process may be on a different time scale and requires further consideration.

CsPbX_3_ quantum dots have been shown to be more ionic in nature and in their interactions with ligands, which suggests an ionic migration mechanism [51]. Indeed, it has been shown that inorganic halide perovskites have high ionic conductivities due to anion migration and the exchange/replacement of the halide ions in these perovskites is facile [50,52,53]. Thus, when mixing two pure, unalloyed perovskite quantum dot solutions, the nanoparticle alloy formation is likely facilitated by anionic migration and to some extent by the free ligands present in the solution [50,51]. The ease and rapidity of the halide exchange/alloying indicates this is thermodynamically favorable.

In this experiment, three different printed layers are investigated, as shown in Figure 1b–d: (1) printing single layer films of pure (CsPbBr_3_ or CsPbI_3_) and/or mixed perovskite CsPbBr_3−x_I_x_ inks, where the mixed perovskites have directly alloyed in solution from different anion nanoparticles, (single-layer printing), (2) printing successive layers of unalloyed CsPbBr_3_ and CsPbI_3_ one on top of the other (inter-layer printing), and (3) printing a thick layer (several layers) of CsPbI_3_ onto a substrate followed by printing a thick layer of CsPbBr_3_ on top of the CsPbI_3_ layer (bi-layer printing).

**Figure 2:**
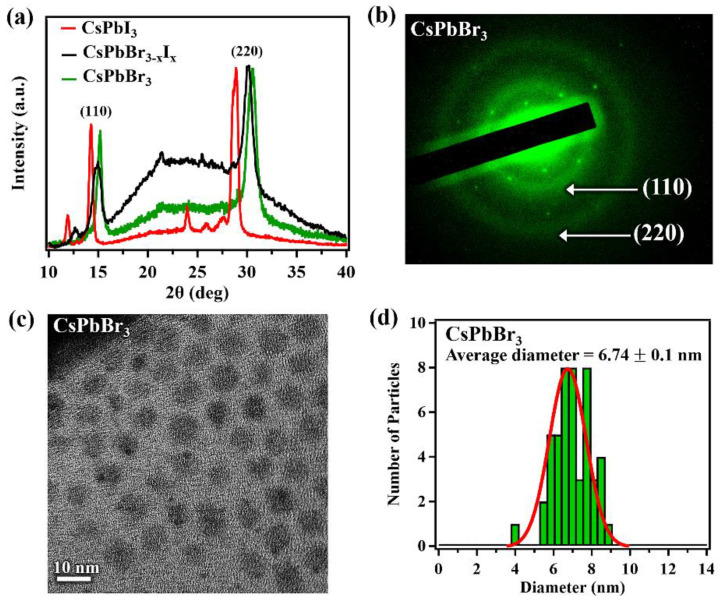
(**a**) the XRD spectra for CsPbI_3_ (red), CsPbBr_3−x_I_x_ (black) and CsPbBr_3_ (green) single-layer printed films; (**b**) SAED of CsPbBr_3_; (**c**) TEM of CsPbBr_3_; and (**d**) nanoparticle size distribution histogram for CsPbBr_3._

The XRD patterns for the single-layer printed films of pure CsPbX_3_ quantum dots and the direct mixed CsPbBr_3−x_I_x_ quantum dots are shown in Figure 2a, indicating a crystal structure in the orthorhombic phase at room temperature in agreement with the literature [54,55,56] and reference card ICSD 28312. The (110) and (220) peaks of the mixed perovskite show a peak shift compared to the peaks of the pure perovskites, which indicates a lattice constant change. The lattice constants for CsPbBr_3_ are a = 8.207 Å, b = 8.355 Å and c = 11.759 Å and for CsPbI_3_ they are a = 8.8561 Å, b = 8.6361 Å and c = 12.4722 Å. For the CsPbBr_3−x_I_x_ the lattice constants are a = 8.5316Å, b = 8.2158 Å and c = 12.1156 Å. The broad feature around 20–25° is most likely due to the residual olelamine and oleic acid used during the synthesis procedure. The SAED pattern, Figure 2b, for CsPbBr_3_ indicates diffuse rings corresponding to the (110) and (220) diffraction planes of the orthorhombic room temperature phase for the CsPbBr_3_ perovskite in strong agreement with the XRD data.

The structure changes, accompanying the nanoparticle mixed anion alloy formation, as characterized by X-ray diffraction (XRD), are consistent with a single phase material. Thus, by mixing two pure perovskites inks we can obtain a completely alloyed solution with a lattice constant, which falls between the pure (unalloyed) perovskites. The absence of peaks representative of CsPbBr_3_ and CsPbI_3_ in the XRD data of CsPbBr_3−x_I_x_ also suggests the absence of CsPbBr_3_ and CsPbI_3_ clusters within the mixed perovskite. Not only does the lattice constant in perovskites change with the changing anion, as is the case here with the alloying of a bromide with an iodide to form a CsPbBr_3−x_I_x_, but we note that in other perovskites, the lattice parameters can change with the nature of the cationic A-site rare earth ion [57].

The crystallite sizes of the printed films for the pure and mixed perovskites were extracted from the XRD profiles using the Scherrer equation [58] and a standard Lorentzian distribution fitting function of the (220) Bragg peak. The crystallite sizes for all CsPbX_3_ films were determined to be roughly 7.1 ± 1.0 nm. This indicates the halide exchange in direct mixing of pure perovskite solutions does not affect the crystallite size significantly. This surprising retention of crystallite size agrees with a study performed by Akkerman et al. [50]. As a comparison to the printed films, the TEM images of CsPbBr_3_ nanoparticle inks were obtained and indicate the particles possess square-like profiles, Figure 2c. The TEM images were used to obtain the size distribution, as shown in Figure 2d, indicating that the average diameter of the nanoparticles is 6.74 ± 0.1 nm, in good agreement with the crystallite size in the films determined with XRD. Interestingly, the XRD peaks of the directly mixed perovskite from a mixed nanoparticle solution, appear closer to the CsPbBr_3_ diffraction peak placement than CsPbI_3_. This indicates that the direct mixed perovskite is heavily weighted towards a large Br anion concentration

**Figure 3:**
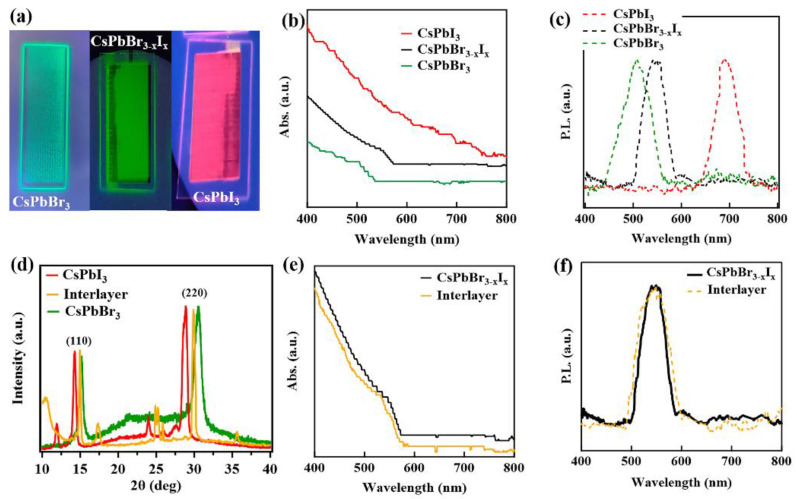
(**a**) image of printed films under UV light, (**b**) optical absorption spectra obtained for printed films of CsPbBr_3_, CsPbI_3_ and alloyed CsPbBr_3−x_I_x_., (**c**) PL spectra for printed films of CsPbBr_3_, CsPbI_3_ and alloyed CsPbBr_3−x_I_x._ (**d**) the XRD spectra CsPbBr_3_ and CsPbI_3_ single-layer printed films, compared to the inter-layer printed film (**e**) the optical absorption spectra and (**f**) PL spectra of alloyed CsPbBr_3−x_I_x_ perovskite single-layer printed film, compared to the inter-layer printed film (denoted interlayer).

The structural changes of the mixed anion alloy nanoparticle is consistent with the changes in the optical properties. The CsPbBr_3−x_I_x_ inks and the printed films are highly luminescent, displaying vivid colors, as shown in Figure 3a, suggesting the optical properties have been tuned to span a broad color spectrum. The optical absorption and background subtracted photoluminescence spectra of the pure CsPbBr3 and CsPbI3 and the direct mixed CsPbBr_3−x_I_x_ quantum dot single-layer printed films are shown in Figure 3b and Figure 3c, respectively. The absorption spectra, Figure 3b, for the pure CsPbBr_3_ film indicates absorption onsets around 499 ± 2 nm and CsPbI_3_ quantum dot printed film indicates strong absorption all over the visible region in agreement with the literature [16,50]. The optical absorption onset, which appears around 542 ± 3 nm for the printed films from alloy CsPbBr_3−x_I_x_ quantum dots (directly alloyed in solution from different anion (Br and I) nanoparticles). This optical absorption for the printed films, from alloy CsPbBr_3−x_I_x_ quantum dots, is located between the absorption onsets of the pure (unalloyed) CsPbBr_3_ and CsPbI_3_ quantum dot films. The photoluminescent spectra, Figure 3c, of pure CsPbBr_3_ and CsPbI_3_ quantum dot printed films shows peaks around 504 ± 1 nm and 691 ± 2 nm, respectively, in agreement with the literature [50]. The photoluminescent peak for the alloyed CsPbBr_3−x_I_x_ quantum dot printed films, following formation from the mixed nanoparticle solutions, appears at 546 ± 4 nm, which again falls between the pure (unalloyed) quantum dot printed films. The absorption and photoluminescent data provide no indication of the clustering of either CsPbBr_3_ or CsPbI_3_ in the direct mixed CsPbBr_3−x_I_x_ single-layer printed films. As the direct mixed CsPbBr_3−x_I_x_ quantum dot printed films exhibit absorption onsets and photoluminescent peaks falling between those of the pure perovskite films and somewhat closer to the CsPbBr_3_ films, in agreement with XRD, these data suggest that anion exchange produces an alloyed single phase perovskite while again supporting the idea that the direct mixed perovskite is heavily weighted towards a higher Br anion concentration.

### 3.2. Nanoparticle Alloying by Anion Exchange across an Inter-Layer Printed Film Interface

In the interest of ascertaining and confirming that alloying is favored across the interface between different anions in the printed films, an inter-layer printing technique was employed by printing alternating layers of pure CsPbBr_3_ and CsPbI_3_, beginning with a layer of CsPbI_3_, as seen in Figure 1. Figure 3d shows a comparison of the XRD data for printed films of the CsPbBr_3_, CsPbI_3_ and an inter-layer printed film prepared by the sequential printing of layers from the pure CsPbBr_3_ and CsPbI_3_ nanoparticle inks. There is a clear indication of alloying across the interlayer. This inter-layer printing method results in interdiffusion across the interface of the CsPbBr_3_ and CsPbI_3_ heterolayers. Interestingly, the (220) and (110) Bragg peaks of the interlayer printed film, as with the direct mixed CsPbBr_3−x_I_x_ single-layer printed films, again fall between the (220) and (110) peaks of the pure (unalloyed) CsPbBr_3_ and CsPbI_3_ films indicating that there is a lattice constant shift due to anion exchange between the inter-diffused layers of CsPbBr_3_ and CsPbI_3_. The optical absorption and photoluminescent spectra of the inter-layer printed films (the denoted inter-layers in Figure 3e,f are compared to those of the direct mixed CsPbBr_3−x_I_x_ single-layer printed quantum dot film, indicating close agreement).

There exists the possibility of limitations to alloying of the heterolayer Br and I anion nanoparticle perovskite inter-layer printed films, which could be consistent with either a higher barrier to interdiffusion or limits to diffusion lengths and rates once printed. These limitations to alloying once the perovskite films are printed were tested using the bi-layer printing method, where a thick layer of CsPbBr_3_ is printed and then a thick layer of CsPbI_3_ is printed on top of the CsPbBr_3_ films. The photoluminescent spectrum, see Supplementary Figure S1, suggests the thicker bi-layer printing method results in unmixed, phase-separated components rather than a uniform alloyed CsPbBr_3−x_I*_x_* perovskite film as seen in the thinner inter-layer printed films, where inter-layer mixing is more uniform (Figure 3d–f) and single-layer printed films made from mixing pure nanoparticle solutions (Figure 3a–c).

In both the single-layer printed alloyed quantum dots film, from mixing pure nanoparticle solutions, and the inter-layer printed film, with mixing occurring at the interfaces, the end result was a material where a final mixed halide state is seen to be strongly preferred. In other words, when mixed together, either in solution or as a heterolayer system, the CsPbBr_3−x_I_x_ perovskites prefer to exist as a single phase mixed alloy rather than remain phase separated, i.e., strongly clustering. The data from single-layer printed film, from mixing pure nanoparticle solutions prior to printing, and the inter-layer printed films, with mixing occurring at the interfaces, suggest that the mixed state acts as a thermodynamic sink and provides a minimum in the energy landscape during the reaction phase of the halide exchange. The thicker bi-layer system is an exception, as seen in supporting information, as this retains some of the separation of the stacked CsPbBr_3_ and CsPbI_3_ films, which could be due to the increased layer thickness and/or rapid solvent evaporation acting as limiting factors in the halide exchange reaction and hetero-layer interdiffusion process. Not all solution based deposition or printing methods lead to the formation of a lead perovskite QD alloy thin film. The cations Cs and formamidinium are not reported to diffuse across the interface in Cs_0.25_FA_0.75_PbI_3_ to CsPbI_3_ heterostructures [59].

From these data it can be inferred that the interlayer printing method has a few advantages compared to the single-layer and bi-layer method. The interlayer printing method results in a completely alloyed film from two distinct parent products, whereas the bi-layer method results in a segregated non-alloyed film with multi-component optical properties. In other words, the interlayer method is capable of producing homogeneous films from multiple source inks that maintain the attractive optical properties of the alloyed materials. Additionally, the ability to print from multiple sources to produce new alloys implies that the interlayer printing method is more versatile and possibly more cost-effective than the single-layer printing method. The interlayer method uses a few parent (CsPbX_3_) solutions (inks), while allowing for various mixed halide films to be produced, thus reducing the total number of perovskite solutions (inks) compared to the single-layer method, which requires a separate ink for each type of film, whether it is a mixed halide or pure halide.

### 3.3. Surface-to-Bulk Core Level Shift

One can imagine, upon the completion of anion (halide) exchange forming a new alloy (CsPbBr_3−x_I_x_), the possibility of electronic surface state and bulk states, which are very different from those in non-alloyed (CsPbBr_3_ or CsPbI_3_) materials, is likely. Additionally, the surface could exhibit an electronic structure different from the bulk, which may be observable in angular-dependent photoemission spectroscopy.

**Figure 4:**
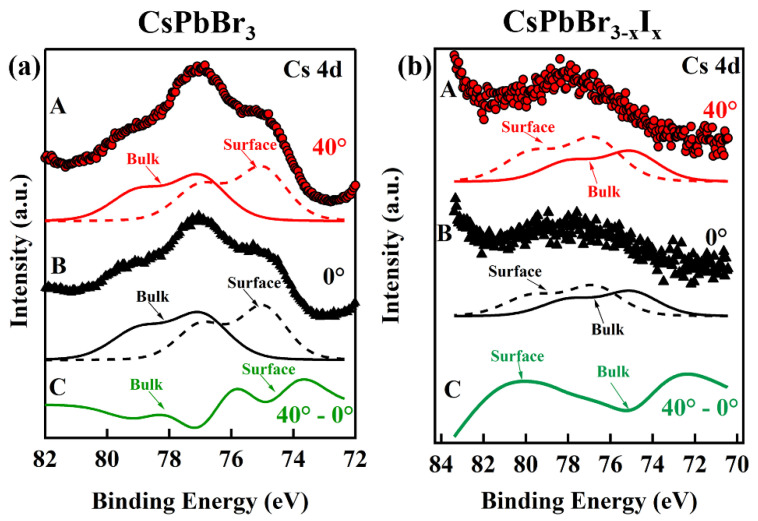
The Cs 4d core level XPS features of the single layer printed (**a**) CsPbBr_3_ and (**b**) CsPbBr_3−x_I_x_ Note: curve A is spectrum taken at an emission takeoff angle of 40°, with respect to the surface normal, the surface and bulk fitted curves of curve A are beneath; curve B is spectrum taken at emission takeoff angle of 0°, i.e., along the surface normal, with the surface and bulk fitted curves of curve B are beneath; curve C is the intensity difference between A and B indicating the surface contribution and the bulk contribution of the surface-to-bulk core level shift.

Interestingly, a surface-to-bulk core level shift [60,61,62] was observed for the Cs 4d core level XPS feature for both the CsPbBr_3_ and the alloy CsPbBr_3−x_I_x_ single-layer printed films, the latter of which forms from the direct mixing of the different anion nanoparticles in solution prior to printing, as shown in Figure 4a and Figure 4b, respectively. In order to increase the surface sensitivity of XPS, the photoemitted electron take-off angle was varied from 0° to 40°. By increasing the take-off angle of the emitted electrons from 0° to 40°, the intensity of a surface contribution will appear to increase in intensity relative to the bulk contribution [60,61]. In Figure 4a,b, curve A (red dots) corresponds to a photoemitted electron takeoff angle of 40° and curve B (black triangles) corresponds to a takeoff angle of 0°, with respect to the surface normal. Beneath each curve, the surface (dashed lines) and bulk (solid lines) contribution fittings are displayed. The intensity difference, curve C, between curve A and curve B, is plotted as a solid green line.

As shown in Figure 4, both films (CsPbBr_3_ and CsPbBr_3−x_I_x_) exhibit increases in their respective photoemission intensity, i.e., surface states, which emerge with increasing take-off angles. If one considers the intensity difference curve, curve C (solid green line), then for CsPbBr_3_ curve C indicates the surface contribution is a broad peak at lower binding energy and the bulk contribution is a dip at higher binding energy. In comparison, curve C for CsPbBr_3−x_I_x_ films indicates the surface contribution is a broad peak at higher binding energy than the bulk contribution (dip) at lower binding energy. The appearance of a surface state at low binding energy in CsPbBr_3_ and at high binding energy in CsPbBr_3−x_I_3_ suggests that the surface terminations are different for the quantum dot films.

The X-ray photoemission (XPS), shown in Supplementary Figure S2, was utilized in order to investigate the composition of the CsPbBr_3−x_I_x_ quantum dot printed films, formed after mixing the different anion nanoparticle solutions. The Cs 3d_5/2_ and 3d_3/2_ core level photoemission peaks are located at 723.8 ± 0.2 eV and 737.88 ± 0.2 eV, respectively. The Pb 4f_7/2_ and 4f_3/2_ core level peaks are located at 138.1 ± 0.1 eV and 142.9 ± 0.2 eV, respectively. While the surface segregation of metallic Pb in lead halide perovskites is a well-established phenomenon [63,64,65,66,67,68], no evidence of Pb^0^ clusters at the film surface was evident in core level photoemission spectra at the Pb core level photoemission features. The XPS spectra of Br shows up as a single broad photoemission peak centered around 68 eV, with an additional feature at higher binding energies due to the Cs 4d core level feature. A modified Voigt function was used to fit the broad Br core level peak and peak positions for Br 3d_5/2_, and 3d_3/2_ were extracted and determined to be 68.1 ± 0.2 eV and 69.6 ± 0.2 eV, respectively. The core level photoemission features of I 3d_5/2_ and I 3d_3/2_ are located at 618.4 ± 0.2 eV and 629.9 ± 0.2 eV, respectively. All of the core level photoemission features for Cs, Pb, I, and Br agree well with values reported in the literature for CsPbBr_3_, CsPbI_3_ and mixed perovskites [34,56,69,70,71]. The photoemission intensities were used to calculate a Br:I atomic ratio of roughly 2.4:0.6, suggesting a Br weighted direct mixed perovskite, CsPbBr_2.4_I_0.6_, in agreement with the XRD, optical absorption spectroscopy, and photoluminescent spectroscopy data. The XPS of the Cs 4d and Pb 4f_7/2_ and 4f_3/2_ core level photoemission features provide further evidence that halide clustering, in the alloy, does not occur, as there are no satellite features that are common in Cs-halide clustered materials [72,73]. Cs-halide free clusters are also very unlikely. The Cs lattice atom can exist in only one oxidation state, in a lattice with large electron affinity anions, suggesting that the surface-to-bulk core level shift is attributable to a large difference in the electronic structure of the surface versus the bulk of the thin films. Furthermore, there is a large separation in the binding energies of the surface versus bulk peaks, confirming the idea that the surface electronic structure is vastly different from the bulk [62].

### 3.4. Transient Recombination Measurements

The presence of defects and trap states in these materials is well known and as there is a surface-to-bulk state transition one could postulate, with confidence, that defects/trap states located near the surface may be different, with respect to the excitonic recombination time, than defect/trap states located within the bulk of the material/film. Indeed, in previous work on these materials the transport data and drift carrier lifetimes indicated regions of fast and slow recombination [9], which suggests the presence of at least two recombination pathways and possibly more. Multiple excitonic recombination pathways and energy loss mechanisms would cause the TRPL profile to broaden along the time axis, especially if these states have a strong influence on the exciton recombination [74]. The origins of multiple recombination lifetimes regarding these materials is somewhat ambiguous; moreover, there seems to be little consensus in the literature on established lifetime ranges.

A list of recent TRPL studies on CsPbBr_3_ and CsPbBr_3−x_I*_x_* along with the decay times and postulated origins for those lifetimes are contained in Table 1. Something to note before discussing the postulated origins is that not all of the studies have listed multiple decay event lifetimes, some have one lifetime and others only two or three. Limiting the fitting equations to one or two term exponential equations results in the data fits being too high in the data and/or too low in the data along the vertical axis. This poses a problem for the correct interpretation of the TRPL data and whether or not those fits (single and two term exponential equations) represent the system correctly. Looking carefully at Table 1, the reader can see that the majority of the postulated origins of transient events can be broken down into the following types: surface states, bulk states, traps states, radiative recombination, and non-radiative recombination. Multiple origins for recombination events across similar systems lead credence to the idea that the fitting equations need to account for more than two events in any given system, especially if said system is capable of multiple energy transitions from the excited state, as has been suggested for inorganic lead halides [75]. Another cause for concern is related to the time-ranges for the various postulated origins, which all seem to overlap, at least in the cases presented in Table 1. It may be more instructive to focus on the surface and bulk state times associated only with CsPbBr_3_. The time constant range for surface states is *τ_surface_* = 1.22–23 ns and for bulk states the range is *τ_bulk_* = 3.55–233 ns, clearly there is some overlap again. If one considers the average of the lifetimes, rather than the time-range, then recombination due to surface states is roughly 12 ns and bulk states is roughly 118 ns. We then can say with some confidence that, typically, *τ_surface_ < τ_bulk_*. As trap states can exist at the surface and in the bulk, it is proposed that there should be four possible decay events that would correspond to surface-trap-, surface-, bulk-trap- and bulk-like states, such that *τ_surface_ < τ_surfacetrap_ < τ_bulk_ < τ_bulktrap_*. In this scenario, it is assumed that trap states (shallow) are long-lived states that do not promote recombination, although it is acknowledged that deep trap states may exist and would promote fast recombination.

**Figure 5:**
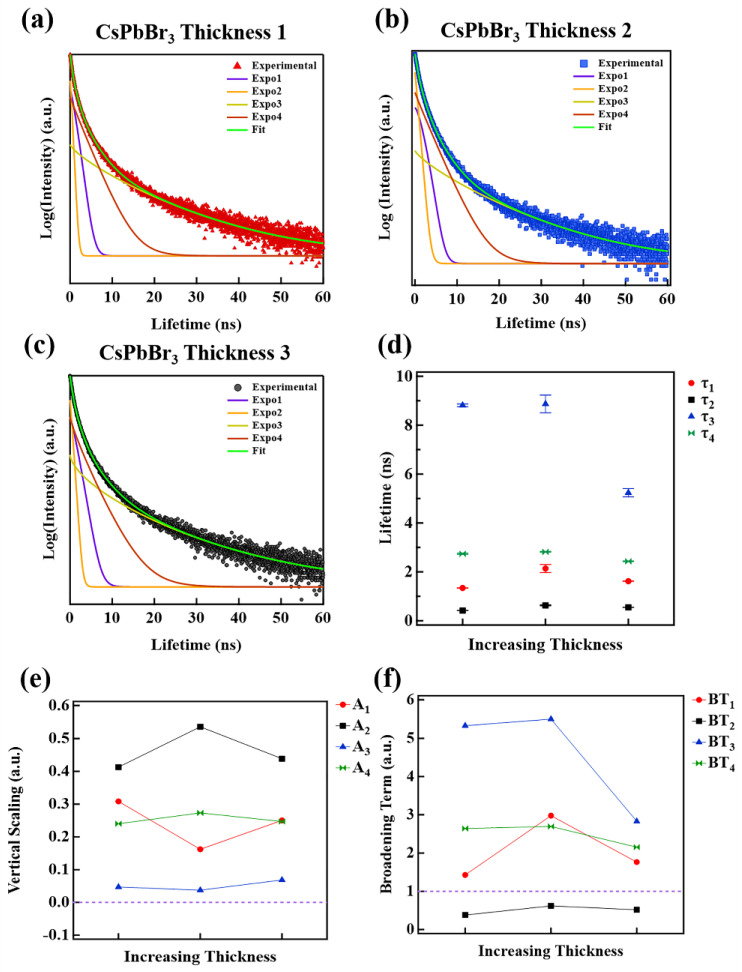
(**a**) TRPL spectra for single-layer method of CsPbBr_3_ films of thickness 1, (**b**) thickness 2 and (**c**) thickness 3, such that thickness 1 < thickness 2 < thickness 3; (**d**) the extracted time constants vs. layer thickness; (**e**) vertical scaling constants vs. layer thickness; and (**f**) broadening term vs. layer thickness.

TRPL measurements for the CsPbBr_3_ films of increasing thickness are shown in Figure 5a–c. The TRPL profiles exhibit a broad exponential decay profile, which suggests that the regular exponential decay functions may not describe the events properly. Indeed single, double and triple regular and stretched exponential functions cannot be used to confidently fit the data. A least squares fitting method was employed with the following quadruple stretched exponential Equation:

(1) $$f\left(t\right)=A_{1}e^{-{\left(\frac{t}{\tau_{1}}\right)}^{\beta_{1}}}+A_{2}e^{-{\left(\frac{t}{\tau_{2}}\right)}^{\beta_{2}}}+A_{3}e^{-{\left(\frac{t}{\tau_{3}}\right)}^{\beta_{3}}}+A_{4}e^{-{\left(\frac{t}{\tau_{4}}\right)}^{\beta_{4}}}+c$$

where *A*_1_–*A*_4_ are the weighted contribution terms, which describe the relative importance of the exponential terms and thus the prevalence of a recombination event. The lifetimes, *τ*_1_–*τ*_4_, correspond to the recombination lifetimes of the exciton recombination events. The power terms, *β*_1_–*β*_4_, influence the horizontal broadening of the exponential terms, representing the vertical shift of Equation (1), rather than the noise floor of the TRPL signal.

The associated recombination lifetimes for the CsPbBr_3_ films where the single-layer printing method is used, are extracted from the fittings using Equation (1) and plotted versus increasing film thickness in Figure 5d. All lifetime curves are distinct, with respect to the error bars, for all sample thicknesses, meaning none of the curves intersect or overlap.

Interestingly, the lifetime curves seem to group into two regions, namely fast and slow recombination time regions, where for fast recombination *τ* < 4.0 ns (includes *τ*_1_*, τ*_2_*,* and *τ*_4_) and for slow recombination *τ* > 4.0 ns (includes *τ*_3_). The lifetime curves in the fast recombination region (*τ*_1_*, τ*_2_ and *τ*_4_) have a mostly constant slope, independent of layer thickness, whereas the lifetime curve in the slow recombination region (*τ*_3_) begins to reduce with increasing thickness. If one considers the average of the lifetimes for each *τ_i_* curve, *τ_avg_*_1_ = 1.70 ns, *τ_avg_*_2_ = 0.53 ns, *τ_avg_*_3_ = 7.64 ns, and *τ_avg_*_4_ = 2.66 ns. In other words, the lifetimes indicate that *τ_avg_*_2_ *< τ_avg_*_1_ *< τ_avg_*_4_ *< τ_avg_*_3_. Surface states are theorized to promote recombination due to the abrupt termination of the crystal lattice [88,89,90], and as such, the fastest lifetimes are likely associated with surface-related recombination. By the convention of the proposed model, where *τ_surface_ < τ_surfacetrap_ < τ_bulk_ < τ_bulktrap_*, the assignment of the average lifetimes would follow as *τ_surface_* = *τ_avg_*_2_; *τ_surfacetrap_* = *τ_avg_*_1_; *τ_bulk_* = *τ_avg_*_4_; *τ_bulktrap_* = *τ_avg_*_3_.

The weighted contributions to the TRPL spectra are described by the *A_i_* terms in Equation (1). The *A_i_* for each exponential subpeak term versus film thickness are plotted in Figure 5e. The *A_i_* indicates, for each *τ_i_*, the overall contribution to the fit function, contained within the decay profiles shown in Figure 5a–c. Furthermore, the *A_i_* term mathematically indicates the extent of vertical stretching or vertical compressing along the intensity axis for each exponential subpeak of Equation (1), such that if *A_i_* > 0.0, a vertical stretching occurs, and if *A_i_* < 0.0, a vertical compression results. All weighting term curves in Figure 5c are greater than 0.0 for all thicknesses, meaning they all contribute to a vertical stretching of the fitting function, Equation (1). For the most part, the weighting term curves do not vary appreciably with increasing film thickness. *A*_2_ (black line-square) and *A*_3_ (blue line, triangle) are well separated from *A*_1_ (red line, circle) and *A*_4_ (green line, hourglass). The weighted term averages are as follows: *A_avg_*_1_ = 0.24, *A_avg_*_2_ = 0.46, *A_avg_*_3_ = 0.05 and *A_avg_*_4_ = 0.25. This would make the average *A_i_* weighting term inequality appear *as A_avg_*_3_ *< A_avg_*_1_ *< A_avg_*_4_ *< A_avg_*_2_. Due to the dominate nature of the surfaces state contributions to the photoluminescence decay profiles [90,91,92,93], it is expected that surface related recombination events should have the largest A_i_ terms on average. Additionally, it is expected that trap related states will have less density compared to their counterpart states (surface and bulk) and should have smaller weighting term contributions accordingly. Applying these guidelines to the weighting terms leads to the following model-based inequality, *A_bulktrap_ <, A_bulk_ < A_surfacetrap_ < A_surface_*, which is similar to the model-based lifetime inequality, *τ_surface_ < τ_surfacetrap_ < τ_bulk_ < τ_bulktrap_*, except the subscripts are in reverse order. If we use the model-based weighting term inequality as a guide, then assignments of the average weighting terms are as follows *A_surface_* = *A_avg_*_2_; *A_surfacetrap_* = *A_avg_*_1_; *A_bulk_* = *A_avg_*_4_; *A_bulktrap_* = *A_avg_*_3_.

The extended broadening along the time axis, as is apparent in the TRPL data of Figure 5a–c, is a major source of errors in most TRPL fittings if left unaccounted for in the fitting function. It is also challenging to establish a single source or physical process responsible for the broadening due to the complexities inherent in the exciton formation and subsequent recombination process. Once an exciton is formed radiative and non-radiative interactions exist and thus associated recombination and charge transfer rates [74]. Non-radiative interactions that may or may not induce recombination can delay or expedite the eventual recombination of the exciton. The temporal delay leads to a broadening or horizontal stretching along the time axis in TRPL spectra. The broadening terms for each exponential subpeak from Equation (1) versus increasing film thickness are plotted in Figure 5f. In this study, the broadening term is defined as follows:

(2) $${\mathrm{BT}}_{i}=\tau_{i}{}^{\beta_{i}}$$

where *τ_i_* is the lifetime term from the TRPL fitting equation, Equation (1), see Figure 5d. *β_i_* is the power of the exponential power in Equation (1). If *BT_i_* is > 1.0 then a horizontal stretching (broadening) occurs, whereas if *BT_i_* < 1.0, then horizontal compression ensues. For all film thicknesses, the *BT*_1_*, BT*_3_, and *BT*_4_ terms are greater than 1.0, suggesting they add to the horizontal broadening, whereas the *BT*_2_ is less than 1.0 for all thicknesses, which means this acts to compress the fitting curve, similar to a fast recombination process with negligible delay before recombination. If we assume that short delay or fast recombination processes contribute less broadening than the slow recombination processes, then a model-based broadening term inequality can be established, so that *BT_surface_ < BT_surfacetrap_ < BT_bulk_ < BT_bulktrap_.* This is similar to the model-based lifetime inequality, *τ_surface_ < τ_surfacetrap_ < τ_bulk_ < τ_bulktrap_*. On average the broadening terms are *BT_avg_*_1_ = 1.97, *BT_avg_*_2_ = 0.51, *BT_avg_*_3_ = 4.38, *BT_avg_*_4_ = 2.48, such that *BT_avg_*_2_ *< BT_avg_*_1_ *< BT_avg_*_4_ *< BT_avg_*_3_. If we use the model-based broadening term inequality as a guide, then the assignments of the average broadening terms are as follows: *BT_surface_* = *BT_avg_*_2_; *BT_surfacetrap_* = *BT_avg_*_1_; *BT_bulk_* = *BT_avg_*_4_; *BT_bulktrap_* = *BT_avg_*_3_.

All of the fitting constants are consistent in their identification of the proposed process. These findings are extremely important in the context of the debate regarding the existence and role of surface and bulk states in the charge transport properties of CsPbX_3_ photovoltaic cells. More measurements are necessary to explore the role of phonon coupling and the chemical nature of the trap states in these materials.

## 4. Conclusions

This study demonstrates the anion exchange of CsPbBr_3_ and CsPbI_3_ solutions/films results in high quality alloyed CsPbBr_3−x_I_x_ nanoparticle textured thin films, through both the direct mixing of nanoparticles in solution and heterolayer printed films, with mixing occurring at the interlayer. The halide exchange in both methods results in a single phase completely alloyed mixed perovskite as confirmed by XRD, optical absorption spectroscopy, photoluminescent spectroscopy, and X-ray photoemission spectroscopy. The mixed halide state acts as a thermodynamic sink, providing a minimum in the energy landscape of the halide anion exchange reaction. Additionally, the electronic structure of the film surface is shown to be very different compared to the bulk through the observation of a surface-to-bulk core level shift, which provides insight on the origins of the excitonic decay process. The success of this work indicates that the rapid prototyping of various perovskite inks and multilayers is realizable. This fundamental fabrication method will lead to a device-by-design routine for promoting next generation solar cell and optical materials/devices, specifically custom lasing devices or LED display screens.

## Figures and Tables

**Table 1:** List of some recent TRPL measurements for CsPbBr_3_ and CsPbBr_3−x_I_x_ materials. Column 1, labeled ‘Material’, indicates the type of material investigated along with reference number. Columns 2, 4 and 6, labeled ‘τ_1_*′*, ‘τ_2_*′* and ‘τ_3_*′*, respectively, are the TRPL lifetimes measured in the associated studies. Columns 3, 5 and 7, labeled ‘origin of τ_1_*′*, ‘origin of τ_2_*′* and ‘origin of τ_3_*′*, respectively, are the physical origin of the lifetimes in columns 2, 4 and 6, respectively, according to the associated reference.

Material [Ref.]	τ_1_	Origin for τ_1_	τ_2_	Origin for τ_2_	τ_3_	Origin for τ_3_
CsPbBr_3_ film NCs [49]	7.9 ns	x				
CsPbBr_2_I film NCs [49]	8.65 ns	x				
CsPbBrI_2_ film NCs [49]	9.25 ns	x				
CsPbBr_3_ (300 K) QDs [76]	3.64 ns	free exciton	8.16 ns	localized exciton		
CsPbBr_3_ (300 K) QDs [77]	6.44 ns	single exciton transition				
CsPbBr_3_ (4K) QDs [77]	355 ps	bright state emission	5.75 ns	lower lying dark state		
CsPbBr_3_ Single Crystal [78]	23 ns	surface state	233 ns	bulk state		
CsPbBr_3_ [79]	7.6 ns	non-radiative recombination	55.9 ns	radiative recombination		
CsPbBr_3_ [80]	1.22 ns	surface state	3.55 ns	bulk state		
CsPbBr/I_3_ [80]	1.7 ns	surface state	11.78 ns	bulk state		
CsPbBr/I_3_ [80]	2.31 ns	surface state	50.39 ns	bulk state		
CsPbBr_3_ [81]	3.8 ns	excitonic recombination	17.6 ns	trap state		
CsPbBr_3_ NCs (4.6nm size) [82]	0.19 ns	trap state	4.1 ns	radiative recombination of single exciton		
CsPbBr_3_ NCs (9.4nm size) [82]	0.43 ns	trap state	6.0 ns	radiative recombination of single exciton		
CsPbBr_3_ NCs (11.4nm size) [82]	11.4 ns	radiative recombination of single exciton				
CsPbBr_3_QDs (220K) [83]	0.193 ns	excitonic recombination				
CsPbBr_3_ [84]	13 ns	x	69 ns	x		
CsPbBr_3_ solution NCs [85]	0.87 ns	x	5.8 ns	x	50 ns	charge trapping
CsPbBr_3_ film NCs [85]	0.93 ns	x	42 ns	charge trapping		
CsPbBr_3_ [86]	4.68 ns	x	22.93 ns	x	140.52 ns	x
CsPbBr/I_3_ [86]	17.09 ns	x	67.71 ns	x	251.63 ns	x
Al:CsPbBr_3_ [86]	5.11 ns	x	34.82 ns	x	139.7 ns	x
Al:CsPbBr/I_3_ [86]	10.52 ns	x	39.97 ns	x	226.2 ns	x
CsPbBr_3_ QDs solution (hexane) [87]	12.3 ns	x	42.4 ns	x	189.0 ns	x
CsPbBr_3_ QDs film (hexane) [87]	1.35 ns	x	5.11 ns	x	19.8 ns	x

## Data Availability

Supplementary Materials contain the additional data pertinent to the paper.

## Supplementary Materials

The following supporting information can be downloaded at: https://www.mdpi.com/article/10.3390/nano12223956/s1, Figure S1: Photoluminescence of the bi-layer CsPbBr_3_/CsPbI_3_ quantum dot printed thin film, the presence of two separate peaks located at roughly 490 nm and 650 nm suggests the presence of segregated CsPbBr_3_ (green line) and CsPbI_3_ (red line), thus confirming the bi-layer printing method results in unmixed layers of CsPbBr_3_/CsPbI_3_; Figure S2: X-ray photoelectron spectroscopy of (a) the Cs 3d core level peaks, (b) Pb 4f core level peaks and (c) the Br 3d core level peaks and Cs 4d core level peaks and (d) the I 3d core level peaks for the direct mixed perovskite CsPbBr_2.4_I_0.6_ quantum dot printed thin films; Figure S3: Optical absorption and photoluminescence profiles for CsPbBr_3_ films, printed with single-layer printing method, of (a) thickness 1, (b) thickness 2 and (c) thickness 3.
