# Supplementary material for: Surface Versus Bulk State Transitions in Inkjet-Printed All-Inorganic Perovskite Quantum Dot Films
Source: Nanomaterials (Basel). 2022 Nov 10;12(22):3956. doi: 10.3390/nano12223956 (PMC9697151; doi:10.3390/nano12223956)
Supplement: Supplementary file 1 [file nanomaterials-12-03956-s001.zip › nanomaterials-2027598-supplementary.pdf]

Supporting Information: Surface *versus* Bulk State Transitions in Inkjet Printed All-Inorganic Perovskite Quantum Dot Films

Thilini K. Ekanayaka[1], Dylan Richmond[2], Mason McCormick[3], Shashank R. Nandyala[4], Halle C. Helfrich[5,6], Alexander Sinitskii[3], John Pikal[4], Carolina C. Ilie[2], Peter A. Dowben[1], and Andrew J. Yost[5,7*]

[1]Department of Physics and Astronomy, University of Nebraska-Lincoln, Lincoln, NE 68588-0299

[2]Department of Physics, State University of New York-Oswego, Oswego, NY 13126-3599

[3]Department of Chemistry, University of Nebraska-Lincoln, Lincoln, NE 68588-0304

[4]Department of Electrical and Computer Engineering, University of Wyoming, Laramie, Wyoming 82071

[5]Department of Physics, Oklahoma State University, Stillwater, Oklahoma, USA 74078

[6]Department of Physics, Pittsburg State University, Pittsburg KS, USA 66762

[7]Oklahoma Photovoltaic Research Institute

[*]Corresponding Author: andrew.yost@okstate.edu

NOTE: Below are transmission electron microscopy images indicating particle size of the $CsPbBr_3$ nanoparticles and additional figures illustrating the X-ray photoemission (XPS) core level features for the printed $CsPbBr_{2.4}I_{0.6}$ nanoparticle textured thin films, through both direct mixing of nanoparticles in solution. There is also photoluminescence spectroscopy data for the thicker bi-layer printed $CsPbBr_3$/$CsPbI_3$ quantum dot thin films and the time dependent alloying of $CsPbBr_{3-x}I_x$ quantum dot solutions. These data were taken at room temperature.. XPS was performed with a SPECS Phoibos 150 hemispherical analyzer using non- monochromatized Al-Kα X-ray radiation and a pass energy of 15 eV in an ultra-high vacuum chamber with a chamber pressure better than 5.0×10^{-10} mbar. The photoluminescent spectroscopy was measured using an Ocean Optics DH-2000-BAL Deuterium-Halogen light source equipped with an Ocean Optics HR4000CG-UV-NIR high resolution spectrometer.

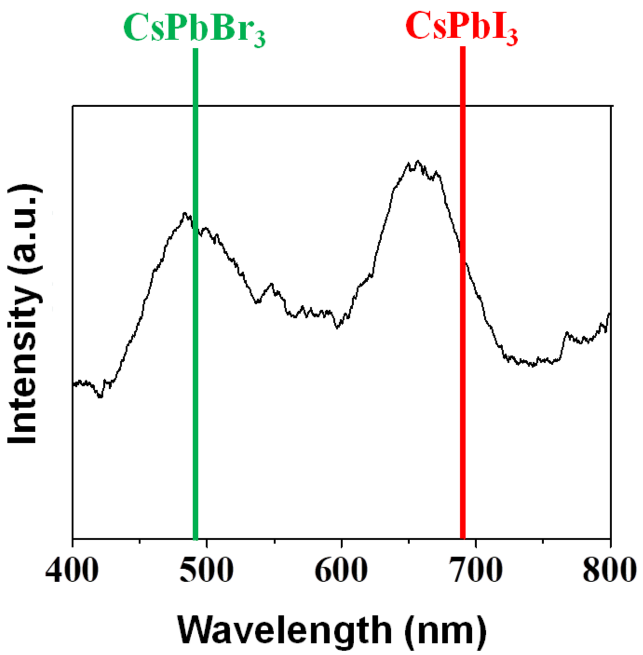

Figure S1: *Photoluminescence of the bi-layer $CsPbBr_3$/$CsPbI_3$ quantum dot printed thin film, the presence of two separate peaks located at roughly 490 nm and 650 nm suggests the presence of segregated $CsPbBr_3$ (green line) and $CsPbI_3$ (red line), thus confirming the bi-layer printing method results in unmixed layers of $CsPbBr_3$/$CsPbI_3$.*

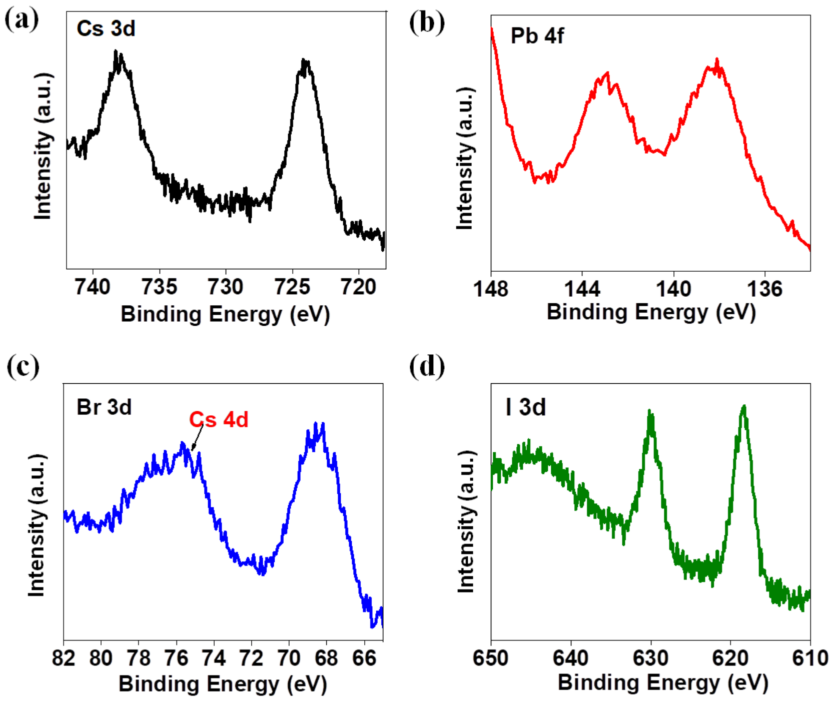

Figure S2: *X-ray photoelectron spectroscopy of (a) the Cs 3d core level peaks, (b) Pb 4f core level peaks, and (c) the Br 3d core level peaks and Cs 4d core level peaks and (d) the I 3d core level peaks for the direct mixed perovskite* $CsPbBr_{2.4}I_{0.6}$ *quantum dot printed thin films.*

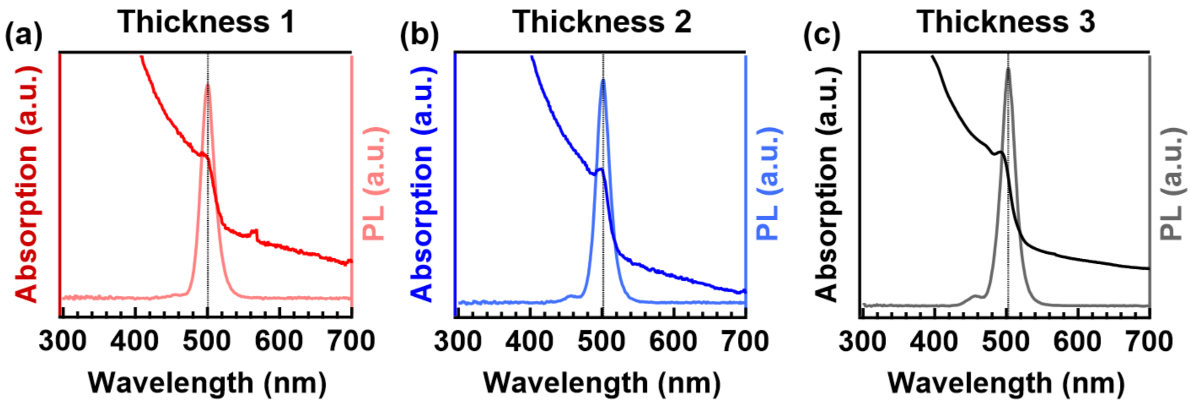

Figure S3: *Optical absorption and photoluminescence profiles for CsPbBr3 films, printed with single-layer printing method, of (a) thickness 1, (b) thickness 2, and (c) thickness 3.The vertical dashed line indicates the position of the PL peak.*
